# The causal relationship between metabolic factors, drinking, smoking and intrahepatic cholangiocarcinoma: a Mendelian randomization study

**DOI:** 10.3389/fonc.2023.1203685

**Published:** 2023-06-22

**Authors:** Shan-shan Qin, Guo-qiang Pan, Qun-bo Meng, Jin-bo Liu, Zi-yu Tian, Shou-jing Luan

**Affiliations:** ^1^ Department of Radiology, Qilu Hospital, Shandong University, Jinan, China; ^2^ Department of General Surgery, Qilu Hospital, Shandong University, Jinan, China; ^3^ Department of Orthopaedical Surgery, Qilu Hospital, Cheeloo College of Medicine, Shandong University, Jinan, China; ^4^ Department of Endocrinology, Weifang People’s Hospital, Weifang, China

**Keywords:** Mendelian randomization, controversial risk factors, ICCA, causal relationship, GWAS data

## Abstract

**Background:**

Intrahepatic cholangiocarcinoma (iCCA) is the second most common primary liver cancer. While multiple risk factors for iCCA have been established, metabolic diseases (obesity, diabetes, NAFLD, dyslipidemia, and hypertension) and other risk factors, including smoking and drinking, are still controversial due to their potential confounders. Here, Mendelian randomization (MR) analysis was performed to identify the causal relationship between them.

**Method:**

In this study, we obtained GWAS data related to exposures from corresponding large genome-wide association studies. Summary-level statistical data for iCCA were obtained from the UK Biobank (UKB). We performed a univariable MR analysis to identify whether genetic evidence of exposure was significantly associated with iCCA risk. A multivariable MR analysis was conducted to estimate the independent effects of exposures on iCCA.

**Results:**

Univariable and multivariable MR analysis based on the large GWAS data indicated that there is little evidence to support the genetic role of metabolic factors, smoking, drinking, and NAFLD in iCCA development (P >0.05). In contrast to most current studies, their impact on iCCA development, if any, might be smaller than we thought. The previous positive results might be due to the comorbidities between diseases and potentially unavoidable confounding factors.

**Conclusion:**

In this MR study, we found no strong evidence to support causal associations between metabolic factors, NAFLD, smoking, drinking, and iCCA risk.

## Introduction

Cholangiocarcinoma (CCA) encompasses a rare group of primary neoplasms arising from the biliary tree. CCAs have traditionally been classified as intrahepatic (iCCA) or extrahepatic (eCCA) based on their anatomical origin within the biliary tree. iCCA, an almost universally lethal malignancy, is the second most common primary liver cancer following hepatocellular carcinoma (HCC), accounting for up to 20% of all hepatic malignancies and 3% of all gastrointestinal malignancies (1, 2). Recent epidemiological reports indicate that the incidence rates of iCCA have been increasing in most countries (3, 4). Compared with lower rates in western countries, the incidence of iCCA is highest in Southeast Asian countries, with worldwide geographical variation directly correlated to region‐specific risk factor profiles (5–8).

Mortality rates from iCCA have increased in most countries in the world, and mortality rates for iCCA are higher than for extrahepatic CCA in most Western countries (4), with 5‐year overall survival (OS) remaining around 9% (9). Most patients have advanced‐stage disease at presentation; diagnosing early-stage iCCA remains a challenge secondary to its silent clinical character (most patients with early-stage disease are asymptomatic). Complete surgical resection remains the only potential cure for iCCA, but only 20%–30% of patients present with resectable disease (10); the mainstream therapeutics for unresectable or metastatic iCCA are palliative chemotherapies; and the median overall survival (OS) is far from satisfactory (11.7 months) (11, 12). Even with only the approved CCA-targeted drugs, pemigatinib-targeting fibroblast growth factor receptor (FGFR) fusion and ivosidenib-targeting isocitrate dehydrogenase (IDH)-1 mutation, the median OS still represents less than 22 months in advanced CCA patients (13). Thus, it is important to identify modifiable risk factors for iCCA.

Several factors with increased risk of iCCA have been identified, including intrahepatic lithiasis, primary sclerosing cholangitis (PSC), congenital abnormalities of the bile ducts, parasite infection, and toxic exposures. Metabolism-associated cholangiocarcinogenic pathways involved in glucose metabolism and glycosylation, as well as altered gut microbiosis, have been implicated in tumorigenesis. Metabolic diseases (obesity, diabetes, NAFLD, and dyslipidemia) and smoking, drinking, and cholelithiasis have long been debated as risk factors for iCCA. Khan and Zhang et al. have systematically summarized the risk factors of iCCA in their review (5, 14). However, controversy has been unsettled regarding the true association between obesity, type 2 diabetes, smoking, and iCCA. For instance, four epidemiologic studies tried to assess the association between obesity and iCCA and found conflicting results (15–18). There are also several studies that tried to explore the association between diabetes and cholangiocarcinoma, but the results for intrahepatic cholangiocarcinoma were also contradictory (16–21). Besides, although most current studies suggest that smoking may represent a risk factor for iCCA, the relative impact of these overlapping diseases on increasing iCCA risk when they co‐occur in the same patient remains an open question. The conclusion seems quite controversial (20–26). It should be noted that most of these risk factors were determined based on case–control studies, and whether such associations are causal is largely unknown. Overall risk factors like metabolic factors, smoking, and drinking are less established and still controversial, with no clear mechanisms that explain their connection to iCCA.

Mendelian randomization (MR) is a method of examining the causal effect of a modifiable exposure on disease by using measured variation in genes of known function in observational data. Because the genotype of an individual is determined at conception and cannot be changed, there is no possibility of reverse causation or confounding bias being responsible for an association between genotype and disease (27). In recent years, many MR studies have emerged to provide clinical evidence (28–30). This proves that MR is a reliable research method to solve some problems, including finding risk factors for diseases.

In our present study, a Mendelian randomization study was used to identify the risk factors, including metabolic factors, drinking, and smoking, for iCCA.

## Methods

### Summarized statistics of risk factors from genome-wide association study

The 19 predominant risk factors can be categorized into four groups, including anthropometric traits, lipidemic traits, glycemic traits, and metabolic diseases.

The instrumental variables (IV) of anthropometric traits were obtained from the Genetic Investigation of ANthropometric Traits (GIANT) consortium. For body mass index (BMI) GWAS, the study included 234,069 European individuals, and the covariates were sex, age, age squared, and principal components. For waist circumference (WC), hip circumference (HC), and waist-to-hip ratio (WHR) GWASs, the participants were 210,088 European individuals, and the study was adjusted for age, age square, and study-specific covariates if necessary (31).

We extracted the GWAS summary statistics of glycemic traits from the Meta-Analyses of Glucose and Insulin-related traits Consortium (MAGIC) (32). There were 281,416 samples, with 70% from European ancestry, in this study, which was adjusted for several covariates, including age, age square, and at all. We only used European summary statistics. We included fasting glucose (FG), fasting insulin (FI), glycated hemoglobin (HbA1c), and 2-hour glucose (2hGlu) post-challenge in the oral glucose tolerance test (OGTT).

There were four lipidemic traits in our study, including total cholesterol (TC), high-density lipoprotein cholesterol (HDL-C), low-density lipoprotein cholesterol (LDL-C), and triglycerides (TG). We obtained summary statistics of the four lipid phenotypes from the Global Lipids Genetics Consortium (GLGC) (33). The GLGC consortium is made up of 188,577 members, with 18,678 of non-European ancestry, and the covariates were sex, age, age squared, BMI, and genotyping chips.

We extracted the GWAS summary statistics of blood pressure, including systolic blood pressure and diastolic blood pressure, from the International Consortium of Blood Pressure (ICBP) (34). This study included 757,601 European-ancestry individuals.

The GWAS summary statistics of T2DM include 26,488 cases and 83,964 controls, with 21,491 cases and 55,647 controls of European ancestry, and this study adjusted for study-specific components (35). For smoking and drinking, we obtained SNPs that had strong associations with smoking behaviors from GWAS and the Sequencing Consortium of Alcohol and Nicotine use (GSCAN), with 249,752 European participants for smoking and drinking (36). Smoking is defined as the average number of cigarettes smoked per day. They included age, sex, age–sex interaction, and the first 10 genetic principal components as covariates and applied genomic control to the GWAS. For NAFLD, we obtained GWAS data from a recent study that included 429,963 individuals (37) (**Table 1**).

**Table 1:** Summary of risk factors.

Exposure	NSNP	Unit	Sample	R2	F	PMID
BMI	97	SD	234069	2.86	70.29	25673413
Body fat percentage	10	SD	65831	1.24	75.13	26833246
Hip circumference	55	SD	210088	1.42	54.03	25673412
Waist circumference	45	SD	210088	1.34	62.02	25673412
Waist-to-hip ratio	34	SD	210088	0.82	49.62	25673412
2h Glucose	15	SD	281416	2.03	364.42	34059833
Fasting Glucose	16	SD	281416	2.08	351.61	34059833
HbA1c	99	SD	281416	4.35	127.94	34059833
Fasting Insulin	42	SD	108557	0.23	50.04	22885924
HDL-C	124	SD	188577	0.13	119.89	24097068
LDL-C	101	SD	188577	0.10	154.41	24097068
TG	72	SD	188577	0.20	147.70	24097068
TC	119	SD	188577	0.11	128.13	24097068
Smoking	28	SD	249752	3.52	314.17	30643251
Type 2 diabetes	35	1-unit in logOR	110452	2.64	83.17	24509480
NAFLD	5	SD	429963	0.16	16572.45	35124268
Drinking	115	SD	462346	0.05	50.28	NA
SBP	769	NA	757601	0.125	64.4	30224653
DBP	790	NA	757601	0.074	65.62	30224653

HbA1c is the glycated hemoglobin; HDL-C is the high-density lipoprotein cholesterol; LDL-C is the low-density lipoprotein cholesterol; TC is the Total cholesterol. TG is the Triglycerides, SBP is systolic blood pressure, DBP is diastolic blood pressure, NAFLD is non-alcoholic fatty liver disease. SD is the standard deviation; log OR is the logarithm of odds ratio.

### Extraction of SNPs associated with intrahepatic cholangiocarcinoma

We searched for iCCA GWAS published before 5 June 2022, with “intrahepatic cholangiocarcinoma” as key words in the GWAS catalog. We found that only one study had been published and was available (38). We also searched the summary statistics of intrahepatic cholangiocarcinoma from the UK Biobank (UKB), and we found the same GWAS data as the GWAS Catalog. The disease code for intrahepatic cholangiocarcinoma in the UK Biobank is “ICD10 C22.1.” This study included 456,348 individuals of European ancestry, and the study adjusted for age, age square, and study-specific covariates.

### Mendelian randomization design and instrumental variable (IV) selection

**Figure 1:**
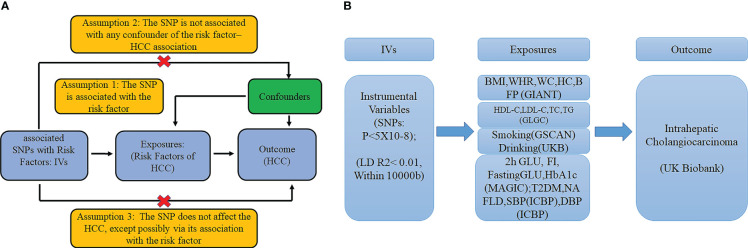
**(A)** The basic assumptions of Mendelian randomization; **(B)** The main design of this study. 2h GLU is the 2-hour glucose after an oral glucose tolerance test. FI is fasting insulin. FG is fasting glucose. BMI is the body mass index; HIP is the hip circumference. WC is the waist circumference. WHR is the waist-to-hip ratio. BFP is the body fat percentage; HbA1c is the glycated hemoglobin; HDL-C is the high-density lipoprotein cholesterol; LDL-C is the low-density lipoprotein cholesterol; and TC is the total cholesterol. TG is the triglycerides. T2DM is type 2 diabetes. SBP is systolic blood pressure, and DBP is diastolic blood pressure. NAFLD is non-alcoholic fatty liver disease.

Mendelian randomization is the use of genetic variants in non-experimental data to make causal inferences about the effect of exposure on an outcome. In MR, genetic variants are used as IVs for assessing the causal effect of the exposure on outcome. The fundamental conditions for a genetic variant to be an IV are summarized as follows: I. The variant is associated with exposure. II. The variant is not associated with any confounders of the exposure–outcome association. III. The variant does not affect the outcome, except possibly *via* its association with the exposure. We selected the significant genetic variants associated with interested exposures from GWAS (significant level p <5 × 10^−8^). And the minor allele frequency of the SNP was >0.01. Then the SNPs used in our study satisfied the linkage disequilibrium (LD, r^2^ <0.01, kb >10,000) in the given genome region, and SNPs with palindromic structure were removed. F statistics (F = beta^2^/se^2^) were used to evaluate the remaining SNPs’ power, so we calculated F statistics for each SNP. SNPs with F statistics <10 were identified as weak instruments and then we excluded them (**Figures 1A, B**).

### Ethics statement

The GWAS summary statistics data used in our study were publicly available, and we obtained informed consent from all participating studies by following the protocols approved by their respective institutional review boards. No separate ethical approval was required for this study.

### Mendelian randomization analysis and sensitivity test

For univariable MR, we used inverse variance weighted (IVW), MR-Egger, and weighted median (WM) to estimate the effect of exposures on outcome. For multivariable MR, we used regression-based IVW. IVW is a method of weighting averages of random variables, where each random variable is weighted by the inverse of its variance. In this study, IVW is the main method adopted in statistical analysis. Besides, the MR-Egger and weighted-median (WM) methods were used as supplements to the IVW method.

We performed the MR-PRESSO global test, outlier test, and distortion test to identify and remove SNPs with horizontal pleiotropy. If any outliers exist, we will restart an evaluation of the causal relationship. The intercept test of MR-Egger and Cochran’s Q test in IVW and the MR Egger model were used to assess pleiotropy and the heterogeneity. In the case of pleiotropy, we prefer to use the MR-Egger. If the P-value in Cochran’s Q test was significant (P <0.05), the WM model would be used to analyze the statistics. Otherwise, a fixed-effects model was used. Furthermore, we conducted a leave-one-out analysis. Moreover, we used online approval to test the statistical power of this study (https://cnsgenomics.shinyapps.io/mRnd/).

Genetic variants associated with exposures at genome-wide significance (p <5 × 10^−8^) were then LD-pruned (distance threshold = 10,000 kb, r^2 = ^0.01) using the clump_data command in the “TwoSampleMR” package in R to identify an independent set of variants to serve as a genetic instrument for exposures. The univariable MR analysis was performed by R packages “Two Sample MR” and “Mendelian randomization.” The multivariable MR analysis was performed by R packages “MVMR” and “Mendelian randomization.” The MR-PRESSO was conducted using the R package “MRPRESSO.” Data visualization was conducted using R software 4.1.1 (https://www.r-project.org/).

## Results

### Univariable MR analysis of risk factors on iCCA

In contrast to some conventional analyses showing a positive association between phenotypes associated with metabolic factors, smoking, and drinking and iCCA risks, the MR analysis indicated no strong evidence to support causality between them for iCCA based on the European population.

#### Metabolic factors

*1.1 Anthropometric traits:* BMI (OR = 1.994, CI = 0.535–7.429, p = 0.304); body fat percentage (OR = 1.791, CI = 0.124–25.942, P = 0.669); hip circumference (OR = 1.401, CI = 0.348–5.645, p = 0.665); waist-to-hip ratio (OR = 1.912, CI = 0.238–15.376, p = 0.542); waist circumference ( OR= 1.943, CI = 0.352-10.734).

*1.2 Blood pressure:* SBP (OR = 1.006, CI = 1.005–1.006, P = 0.784); DBP (OR = 1.007, CI = 1.006–1.007, p = 0.845).

*1.3 Glycemic traits:* 2h glucose (OR = 1.183, CI = 0.351–3.982, p = 0.788); fasting glucose (OR = 0.826, CI = 0.064–10.615, p = 0.883); HbA1c (OR = 1.130, CI = 0.170–7.529, p = 0.911); fasting insulin (OR = 4.002, CI = 0.157–102.318, p = 0.373); type 2 diabetes (OR = 0.671, CI = 0.363–1.242, p = 0.204).

*1.4 Lipidemic traits:* HDL-C (OR = 0.731, CI = 0.385–1.390, p = 0.339); LDL-C (OR = 1.232, CI = 0.694–2.187, p = 0.475); TG (OR = 1.053, CI = 0.477–2.324, p = 0.898); TC (OR = 1.029, CI = 0.577–1.836, p = 0.923).

*1.5 NAFLD:* (OR = 0.739, CI = 0.637–0.856, p = 0.223).

#### Smoking and drinking

Smoking (OR = 0.751, CI = 0.285–1.976, p = 0.562), drinking (OR = 1.508, CI = 0.918–2.475, p = 0.505) (**Table 2**).

**Table 2:** The effect estimates, test of heterogeneity and test of pleiotropy of Risk Factors on iCCA.

Exposure	NSNP	MR methodology	Effect Estimates iCCA				Test of heterogeneity		Test of pleiotropy	
			OR	95%LCI	95%UCI	P value	Cochrane Q test	Phetero- geneity	MR Egger intercept	Ppleio- tropy
**BMI**	79	IVW	1.994	0.535	7.429	0.304	62.818	0.894		
		MR Egger	0.087	0.002	4.324	0.224	60.032	0.923	0.090	0.099
		Weighted median	1.636	0.240	11.158	0.611				
**Body fat percentage**	10	IVW	1.791	0.124	25.942	0.669	7.984	0.536		
		MR Egger	0.001	6.11x10^-9^	336.641	0.330	6.644	0.575	-0.022	0.964
		Weighted median	2.003	0.056	70.949	0.710				
**Hip circumference**	54	IVW	1.401	0.348	5.645	0.665	5.046	0.538		
		MR Egger	5.858	0.087	392.808	0.333	0.123	0.726	0.263	0.280
		Weighted median	2.274	0.259	19.957	0.754				
**Waist circumference**	44	IVW	1.943	0.352	10.734	0.446	37.421	0.711		
		MR Egger	3.063	0.007	1.152 x10^3^	0.717	37.397	0.673	-0.013	0.878
		Weighted median	1.968	0.153	25.290	0.593				
**Waist-to-hip ratio**	33	IVW	1.912	0.238	15.376	0.542	32.096	0.462		
		MR Egger	10.859	0.002	4.977x10^4^	0.583	31.917	0.421	-0.046	0.679
		Weighted median	8.091	0.394	166.192	0.197				
**SBP**	751	IVW	1.006	1.005	1.006	0.784	765.334	0.341		
		MR Egger	0.983	0.980	0.985	0.751	765.121	0.333	0.007	0.648
		Weighted median	0.981	0.980	0.982	0.581				
**DBP**	790	IVW	1.007	1.006	1.007	0.845	807.345	0.317		
		MR Egger	0.946	0.937	0.956	0.540	806.768	0.314	0.453	
		Weighted median	0.966	0.962	0.970	0.544				
**NAFLD**	5	IVW	0.739	0.637	0.856	0.223	1.627	0.804		
		MR Egger	0.764	0.568	1.029	0.666	1.622	0.654		
		Weighted median	0.684	0.556	0.843	0.175				
**2h Glucose**	14	IVW	1.183	0.351	3.982	0.788	12.776	0.465		
		MR Egger	5.553	0.221	139.237	0.318	11.742	0.467	-0.117	0.329
		Weighted median	1.210	0.230	6.350	0.824				
**Fasting Glucose**	29	IVW	0.826	0.064	10.615	0.883	24.036	0.680		
		MR Egger	4.409	0.005	4.16x10^3^	0.674	23.769	0.643	-0.043	0.609
		Weighted median	0.606	0.013	27.638	0.795				
**HbA1c**	94	IVW	1.130	0.170	7.529	0.911	71.968	0.948		
		MR Egger	0.681	0.013	34.431	0.848	71.876	0.940	0.010	0.762
		Weighted median	0.692	0.015	31.640	0.848				
**Fasting Insulin**	42	IVW	4.002	0.157	102.318	0.373	46.330	0.262		
		MR Egger	0.004	3.05x10^-7^	44.141	0.249	43.701	0.317	0.125	0.129
		Weighted median	7.818	0.093	660.411	0.363				
**Type 2 diabetes**	24	IVW	0.671	0.363	1.242	0.204	19.388	0.679		
		MR Egger	4.191	0.305	57.550	0.295	17.402	0.741	-0.193	0.173
		Weighted median	0.560	0.238	1.315	0.183				
**HDL-C**	122	IVW	0.731	0.385	1.390	0.339	115.125	0.633		
		MR Egger	0.581	0.176	1.917	0.375	114.925	0.614	0.013	0.656
		Weighted median	0.750	0.272	2.068	0.578				
**LDL-C**	95	IVW	1.232	0.694	2.187	0.475	99.902	0.319		
		MR Egger	0.995	0.403	2.455	0.991	99.497	0.303	0.017	0.540
		Weighted median	0.894	0.364	2.196	0.807				
**TG**	70	IVW	1.053	0.477	2.324	0.898	72.385	0.367		
		MR Egger	0.969	0.268	3.501	0.962	72.356	0.336	0.005	0.869
		Weighted median	1.043	0.311	3.505	0.945				
**TC**	114	IVW	1.029	0.577	1.836	0.923	111.521	0.522		
		MR Egger	1.697	0.664	4.335	0.272	109.758	0.542	-0.034	0.187
		Weighted median	1.428	0.517	3.939	0.501				
**Smoking**	28	IVW	0.751	0.285	1.976	0.562	27.368	0.444		
		MR Egger	0.732	0.127	4.208	0.729	27.367	0.390	0.002	0.972
		Weighted median	1.471	0.344	6.280	0.192				
**Drinking**	112	IVW	1.508	0.918	2.475	0.505	105.130	0.639		
		MR Egger	11.127	0.010	1.25x10^4^	0.108	102.949	0.670	-0.054	0.143
		Weighted median	3.592	0.205	63.008	0.263				

NSNP is the number of single nucleotide polymorphism; OR is the odds ratio; 95%LCI is the lower limit of 95% confidence interval; 95%UCI is the upper limit of 95% confidence interval; P value is the p-value of OR; Pheterogeneity is the p-value of Cochrane’s Q value in heterogeneity test; Ppleiotropy is the p-value of MR-Egger intercept. IVW is IVW with a fixed effects model.

### Multivariable MR analysis of risk factors on iCCA

A multivariable MR analysis was conducted to estimate the independent effects of risk factors on iCCA. No significant association was also observed when using exposure-associated SNPs.

#### Metabolic factors

*1.1 Anthropometric traits:* BMI (OR = 2.056, CI = 0.496–8.524, p = 0.321); Body fat percentage (OR = 2.156, CI = 0.148–31.421, p = 0.574); hip circumference (OR = 0.996, CI = 0.225–4.402, p = 0.996); waist circumference (OR = 1.701, CI = 0.262–11.060, p = 0.578).

*1.2 Blood pressure:* SBP (OR = 1,02, CI = 0.975–1.058, p = 0.467); DBP (OR = 1.002, CI = 0.934–1.074, p = 0.965).

*1.3 Glycemic traits:* 2h glucose (OR = 1.271, CI = 0.369–4.385, p = 0.704); fasting glucose (OR = 0.594, CI = 0.046–7.681, p = 0.690); HbA1c (OR = 0.992, CI = 0.116–8.480, p = 0.994); fasting insulin (OR = 2.022, CI = 0.090–45.595, p = 0.658); type 2 diabetes (OR = 0.685, CI = 0.371–1.265, p = 0.227).

*1.4 Lipidemic traits:* HDL-C (OR = 0.703, CI = 0.355–1.392, p = 0.312); LDL-C (OR = 1.241, CI = 0.659–2.337, p = 0.504); TG (OR = 1.247, CI = 0.560–2.775, p = 0.589); TC (OR = 0.984, CI = 0.518–1.873, p = 0.962).

*1.5 NAFLD* (OR = 0.739, CI = 0.455–1.199, p = 0.221).

#### Smoking and drinking

**Figure 2:**
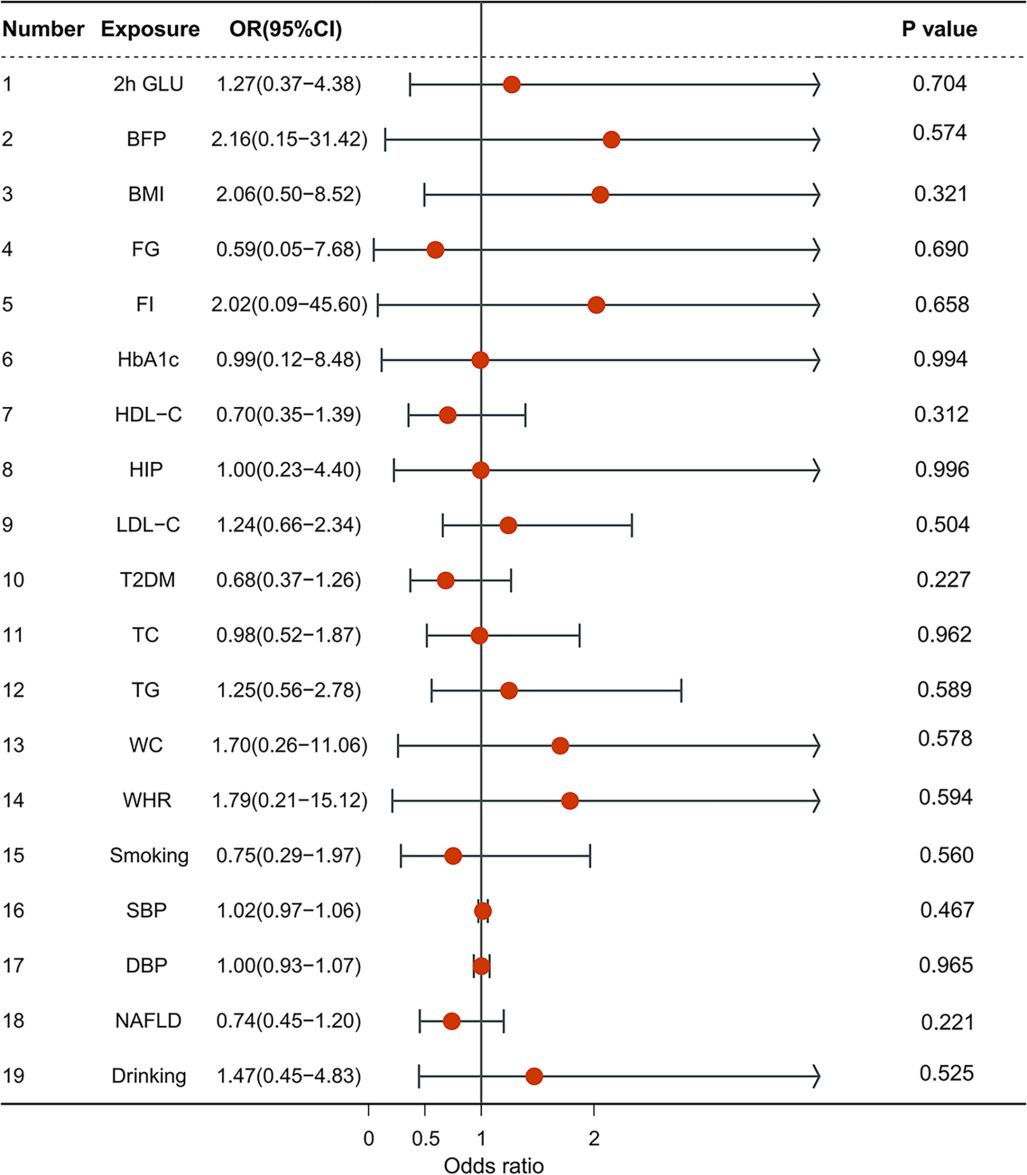
The forest plot of multivariable Mendelian randomization results. OR is the odds ratio; 95% LCI is the lower limit of the 95% confidence interval; 95% UCI is the upper limit of the 95% confidence interval; SD is the standard deviation; and log OR is the logarithm of the odds ratio.

Smoking (OR = 0.751, CI = 0.287–1.966, p = 0.560), drinking (OR = 1.471, CI = 0.448–4.831, p = 0.525) (**Figure 2**).

We observed that the confidence interval for exposures was wide, which was caused by the relatively low sample size. Therefore, it cannot be ruled out that there would be weak connections between metabolic factors, smoking, drinking, and iCCA. Another possibility of null findings observed in our MR analyses could be explained by the low proportion of variances in some exposures (F statistics <100).

### Sensitivity analysis

The heterogeneity and pleiotropy of results had not been tested in this study. MR-Egger intercept represented the average level of pleiotropy of all SNPs associated exposure. No significant horizontal pleiotropic effects were detected in the MR Egger test (for the intercept of MR Egger, all P-values were greater than 0.1). All the results of these exposures were MR-PRESSO corrected if outliers were detected. The statistical power of these exposures was 100%.

## Discussion

iCCA comprises approximately 10% of all primary liver cancers, making it the second most common primary hepatic malignancy after hepatocellular carcinoma (HCC) (39). As well-established risk factors for most kinds of cancers, metabolic diseases (obesity, diabetes, NAFLD, dyslipidemia, and hypertension), smoking, and drinking have been the subject of a long debate as to whether they are risk factors for iCCA. Several observational studies have demonstrated that patients with them have a significantly increased risk of iCCA, while many studies have also reported no association.

In our population-based Mendelian randomization study, we estimated the causal relationship between obesity, type 2 diabetes, NAFLD, hypertension, smoking, drinking, and iCCA based on several large-scale GWAS studies. To make our study more complete, we included glycemic phenotypes associated with type 2 diabetes and anthropometric traits associated with obesity. We provided novel insights into how obesity, type 2 diabetes, dyslipidemia, hypertension, NAFLD, smoking, and drinking might not be associated with iCCA.

Obesity is a global epidemic that has a complex association with many cancers. As the most measured marker for obesity, BMI has been more and more extensively investigated. The data on the relationship between obesity and iCCA is conflicting and not consistent. Menon et al. (19) found a strong positive association between increasing BMI and iCCA, including 4,287 cases in the UK. On the contrary, Welzel et al. and Chaiteerakij et al., who conducted the study on the Danish population and the US population, both assessed the relationship between obesity and iCCA and both found no significant association between obesity and iCCA (16, 18). A large prospective cohort study of people in the U.S. found that a higher risk of iCCA was associated with BMI at age 18 but not at ages 35 and 50. However, the sample size was limited for their analysis, and most results were not significant (40). Another MR study identified an association between 26 risk factors, including metabolic factors, drinking, and smoking. They found that gallstones and liver fat accumulation were two risk factors for CCA (41). Our MR analysis supported that there was no direct causation between obesity and iCCA since none of the obesity-related indices can contribute to the iCCA risk (BMI, WHR, and BFP). Considering that obesity can alter levels of adipokines, pro-tumorigenic lipids, and metabolites, and since liver fat accumulation is an established causal risk factor in previous studies, it might be reasonable that the previously observed association between obesity and iCCA could be mediated by liver fat accumulation. This may be the reason why this study shows that there is no causal relationship between obesity and iCCA.

T2DM is also a recognized risk factor for cancer. Over the decades, several cohort studies have identified associations between type 2 diabetes and increased cancer risk (42). Characteristics of type 2 diabetes include abnormal glucose and lipid metabolism, which might progress to hyperinsulinemia and insulin resistance. These changes promote hepatocyte proliferation and inhibit cellular apoptosis, which could induce iCCA. Studies that assessed the relationship between type 2 diabetes and iCCA reported different findings and conflicting conclusions. Petrick et al. performed a US population-based study and observed that type 2 diabetes is associated with iCCA (20). Palmer et al. conducted a meta-analysis and then also found that type 2 diabetes was significantly associated with iCCA (21). Similar conclusions can also be obtained from Welzel et al.’s research (15, 16). Nevertheless, several studies obtained different results. One case–control study based on the UK population conducted by Grainge et al. found an association between diabetes and both cholangiocarcinoma and gallbladder cancer, but that association was not statistically significant with cholangiocarcinoma when analyzing the subgroup data (23). Another study based on the Taiwanese population found that although there was an association between diabetes and cholangiocarcinoma, the association was non-significant (43). Similar conflicting results have been observed from other studies on type 2 diabetes and iCCA based on East Asian populations, including Chinese and Japanese (44). In this MR study, the circumstances of T2DM and its associated indices are similar to those of obesity, where the MR analysis found no strong evidence to support associations between T2DM, including several glycemic traits, and iCCA as well. The largest meta-analysis did not find an association between hypertension and CCA, and our MR analysis supported it (28).

Non-alcoholic fatty liver disease (NAFLD) is causing a rise in the prevalence of hepatocellular carcinoma. On the contrary, the relationship between NAFLD and iCCA is still unclear, with conflicting data being reported. A multicenter international study showed that NAFLD and its most aggressive phenotype (non-alcoholic steatohepatitis, NASH) are held responsible for the increasing incidence of iCCA and its prognostic role (45). NAFLD has been associated with iCCA, but such an association was not confirmed in this MR study. Such results can be explained by the existing confounders as well.

Lipid metabolism plays a vital role in the stability of the cell membrane and energy supply, thereby impacting cell growth and proliferation *via* multiple signaling pathways. Lipids and cholesterol can be exploited by most types of cancer to meet their increased energy demands. Cancer cells even possess some adipocyte characteristics, storing excess lipids in the form of lipid droplets, which supply energy to promote their expansion and metastasis. Although most current research deems that serum lipid profiles are associated with cancer, the relationship between serum lipid profiles and cancers at specific sites has not been confirmed. This notion is supported by the lack of a causal association between serum lipid profiles and iCCA. The largest meta-analysis indicated that both smoking and drinking were risk factors for iCCA, while our results suggested that such associations might not be directly causal. The reason for this contradictory result may be that smoking could increase the risk of NAFLD, and it is no surprise that there was an association between smoking and iCCA in that study (28). Additionally, it has been well established that alcohol intake can damage the liver and cause lipid accumulation. Thus, there might be no direct causal link between alcohol intake and iCCA. Although the proportion of cases included in the UKB is relatively low, the probability of a false negative relationship between risk factors and iCCA should be low after rigorous IV selection and sensitivity testing.

In conclusion, this study is the first to use MR to evaluate the causal relationship between obesity, type 2 diabetes, NAFLD, hypertension, dyslipidemia, smoking, drinking, and iCCA, and it found that there is little evidence to support the genetic role of them in iCCA development. It provided a novel insight into current unsettled problems in iCCA: in contrast to most current studies, the impact of type 2 diabetes, obesity, and smoking on iCCA development, if any, might be smaller than we thought. The previous positive results might be due to the comorbidities between diseases and potentially unavoidable confounding factors. However, there are several limitations to our study (1): The proportion of iCCA patients in UKB is relatively low and could compromise statistical power, failing to detect a true causal relationship. Therefore, the 95% confidence intervals of our results were relatively wide, and potential associations of obesity, type 2 diabetes, and smoking with iCCA cannot be ruled out. (2) Another factor that influences the results is pleiotropy; we performed MR Egger and MR PRESSO to reduce the pleiotropy, and the Egger regression did not suggest the possibility of pleiotropic effects. (3) Our study was based on people of European ancestry, and it is hard to consider whether it can be generalized to other populations. (4) Anthropometric traits and several metabolic factors differ between the sexes and races, which may also lead to bias in the MR analysis. However, currently, most of the GWAS data used for MR analysis is mixed-sex and Eastern-Asian ancestry data. Further MR studies are needed to address this problem. (5) In this study, the included metabolism-related risk factors and metabolism-related diseases are relatively insufficient and need to be improved in future analyses. Anyway, further investigations should be carried out to verify these findings and hypotheses.

## Data availability statement

The original contributions presented in the study are included in the article/supplementary material. Further inquiries can be directed to the corresponding author.

## Ethics statement

The GWAS summary statistics data used in our study were publicly available, which obtained informed consent from all participating studies by following the protocols approved by their respective institutional review boards. No separate ethical approval was required for this study.

## Author contributions

Study concept and design: S-JL. Analysis of data: G-QP. Quality assessment: S-JL. Manuscript draft: S-SQ, Z-YT, Q-BM, and J-BL. All authors read and approved the final manuscript. All authors contributed to the article and approved the submitted version.

## References

[1] Siegel RL Miller KD Fuchs HE Jemal A. Cancer statistics, 2022. CA Cancer J Clin (2022) 72(1):7–33. doi: 10.3322/caac.21708 35020204

[2] Beal EW Tumin D Moris D Zhang XF Chakedis J Dilhoff M. Cohort contributions to trends in the incidence and mortality of intrahepatic cholangiocarcinoma. Hepatobiliary Surg Nutr (2018) 7(4):270–6. doi: 10.21037/hbsn.2018.03.16 PMC6131266 30221154

[3] Saha SK Zhu AX Fuchs CS Brooks GA. Forty-year trends in cholangiocarcinoma incidence in the U.S.: intrahepatic disease on the rise. Oncologist (2016) 21(5):594–9. doi: 10.1634/theoncologist.2015-0446 PMC4861366 27000463

[4] Bertuccio P Malvezzi M Carioli G Hashim D Boffetta P El-Seraget al H. Global trends in mortality from intrahepatic and extrahepatic cholangiocarcinoma. J Hepatol (2019) 71(1):104–14. doi: 10.1016/j.jhep.2019.03.013 30910538

[5] Khan SA Tavolari S Brandi G. Cholangiocarcinoma: epidemiology and risk factors. Liver Int (2019) 39(Suppl 1):19–31. doi: 10.1111/liv.14095 30851228

[6] Florio AA Ferlay J Znaor A Ruggieri D Alvarez C Laversanne M. Global trends in intrahepatic and extrahepatic cholangiocarcinoma incidence from 1993 to 2012. Cancer (2020) 126(11):2666–78. doi: 10.1002/cncr.32803 PMC7323858 32129902

[7] Altekruse SF Petrick JL Rolin AI Cuccinelli J Zou ZH Tatalovich Z. Geographic variation of intrahepatic cholangiocarcinoma, extrahepatic cholangiocarcinoma, and hepatocellular carcinoma in the united states. PLoS One (2015) 10(3):e0120574. doi: 10.1371/journal.pone.0120574 25837669 PMC4383424

[8] Vithayathil M Khan SA. Current epidemiology of cholangiocarcinoma in Western countries. J Hepatol (2022) 77(6):1690–8. doi: 10.1016/j.jhep.2022.07.022 35977611

[9] Yao KJ Jabbour S Parekh N Lin Y Moss RA. Increasing mortality in the united states from cholangiocarcinoma: an analysis of the national center for health statistics database. BMC Gastroenterol (2016) 16(1):117. doi: 10.1186/s12876-016-0527-z 27655244 PMC5031355

[10] Endo I Gonen M Yopp AC Dalal K Zhou Q Klimstra D. Intrahepatic cholangiocarcinoma: rising frequency, improved survival, and determinants of outcome after resection. Ann Surg (2008) 248(1):84–96. doi: 10.1097/SLA.0b013e318176c4d3 18580211

[11] Dhanasekaran R Hemming AW Zendejas I George T Nelson DR Soldevila-Pico C. Treatment outcomes and prognostic factors of intrahepatic cholangiocarcinoma. Oncol Rep (2013) 29(4):1259–67. doi: 10.3892/or.2013.2290 PMC3621732 23426976

[12] Valle J Wasan H Palmer DH Cunningham D Anthoney A Maraveyas A. Cisplatin plus gemcitabine versus gemcitabine for biliary tract cancer. N Engl J Med (2010) 362(14):1273–81. doi: 10.1056/NEJMoa0908721 20375404

[13] Saha SK Gordan JD Kleinstiver BP Vu P Najem M Yeo JC. Isocitrate dehydrogenase mutations confer dasatinib hypersensitivity and SRC dependence in intrahepatic cholangiocarcinoma. Cancer Discov (2016) 6(7):727–39. doi: 10.1158/2159-8290.Cd-15-1442 PMC5458737 27231123

[14] Zhang H Yang T Wu M Shen F. Intrahepatic cholangiocarcinoma: epidemiology, risk factors, diagnosis and surgical management. Cancer Lett (2016) 379(2):198–205. doi: 10.1016/j.canlet.2015.09.008 26409434

[15] Welzel TM Graubard BI El-Serag HB Shaib Y Hsing A Davila J. Risk factors for intrahepatic and extrahepatic cholangiocarcinoma in the united states: a population-based case-control study. Clin Gastroenterol Hepatol (2007) 5(10):1221–8. doi: 10.1016/j.cgh.2007.05.020 PMC2083573 17689296

[16] Welzel TM Mellemkjaer L Gloria G Sakoda L Hsing A Ghormli L. Risk factors for intrahepatic cholangiocarcinoma in a low-risk population: a nationwide case-control study. Int J Cancer (2007) 120(3):638–41. doi: 10.1002/ijc.22283 17109384

[17] Choi J Ghoz HM Peeraphatdit T Baichoo E Addissie B Harmsen WS. Aspirin use and the risk of cholangiocarcinoma. Hepatology (2016) 64(3):785–96. doi: 10.1002/hep.28529 PMC5995727 26940227

[18] Chaiteerakij R Yang JD Harmsen WS Slettedahl SW Mettler TA Fredericksen ZS. Risk factors for intrahepatic cholangiocarcinoma: association between metformin use and reduced cancer risk. Hepatology (2013) 57(2):648–55. doi: 10.1002/hep.26092 PMC3565026 23055147

[19] Menon S Mathew R. Association between metabolic syndrome and hepatobiliary cancers: a case-control study. Indian J Gastroenterol (2019) 38(1):61–8. doi: 10.1007/s12664-018-0925-y 30628006

[20] Petrick JL Yang B Altekruse SF Dyke AL Koshiol J Graubardet B. Risk factors for intrahepatic and extrahepatic cholangiocarcinoma in the united states: a population-based study in SEER-Medicare. PLoS One (2017) 12(10):e0186643. doi: 10.1371/journal.pone.0186643 29049401 PMC5648218

[21] Palmer WC Patel T. Are common factors involved in the pathogenesis of primary liver cancers? a meta-analysis of risk factors for intrahepatic cholangiocarcinoma. J Hepatol (2012) 57(1):69–76. doi: 10.1016/j.jhep.2012.02.022 22420979 PMC3804834

[22] McGee EE Jackson SS Petrick JL Dyke A Adami H Albanes D. Smoking, alcohol, and biliary tract cancer risk: a pooling project of 26 prospective studies. J Natl Cancer Inst (2019) 111(12):1263–78. doi: 10.1093/jnci/djz103 PMC6910180 31127946

[23] Grainge MJ West J Solaymani-Dodaran M Aithal GP Card TR. The antecedents of biliary cancer: a primary care case-control study in the united kingdom. Br J Cancer (2009) 100(1):178–80. doi: 10.1038/sj.bjc.6604765 PMC2634685 19018260

[24] Ye XH Huai JP Ding J Chen YP Sun XC. Smoking, alcohol consumption, and the risk of extrahepatic cholangiocarcinoma: a meta-analysis. World J Gastroenterol (2013) 19(46):8780–8. doi: 10.3748/wjg.v19.i46.8780 PMC3870528 24379600

[25] Kan HP Huang YQ Tan YF Zhou J. Meta-analysis of alcohol consumption and risk of extrahepatic bile system cancer. Hepatol Res (2011) 41(8):746–53. doi: 10.1111/j.1872-034X.2011.00831.x 21794037

[26] Shaib YH El-Serag HB Nooka AK Thomas M Brown T Patt P. Risk factors for intrahepatic and extrahepatic cholangiocarcinoma: a hospital-based case-control study. Am J Gastroenterol (2007) 102(5):1016–21. doi: 10.1111/j.1572-0241.2007.01104.x 17324130

[27] Davey Smith G Hemani G. Mendelian randomization: genetic anchors for causal inference in epidemiological studies. Hum Mol Genet (2014) 23(R1):R89–98. doi: 10.1093/hmg/ddu328 PMC4170722 25064373

[28] Chen L Yang H Li H He C Yang L Lv G. Insights into modifiable risk factors of cholelithiasis: a mendelian randomization study. Hepatology (2022) 75(4):785–96. doi: 10.1002/hep.32183 PMC9300195 34624136

[29] Pan GQ Yang CC Shang XL Dong ZR Li T. The causal relationship between white blood cell counts and hepatocellular carcinoma: a mendelian randomization study. Eur J Med Res (2022) 27(1):278. doi: 10.1186/s40001-022-00900-y 36471350 PMC9724280

[30] Larsson SC Gill D. Association of serum magnesium levels with risk of intracranial aneurysm: a mendelian randomization study. Neurology (2021) 97(4):e341–4. doi: 10.1212/wnl.0000000000012244 PMC8362358 34158381

[31] Shungin D Winkler TW Croteau-Chonka DC Ferreira T Locke E Mägi R. New genetic loci link adipose and insulin biology to body fat distribution. Nature (2015) 518(7538):187–96. doi: 10.1038/nature14132 PMC4338562 25673412

[32] Chen J Spracklen CN Marenne G Varshney A Corbin L Luan JA. The trans-ancestral genomic architecture of glycemic traits. Nat Genet (2021) 53(6):840–60. doi: 10.1038/s41588-021-00852-9 PMC7610958 34059833

[33] Willer CJ Schmidt EM Sengupta S Peloso GM Gustafsson S Kanoni S. Discovery and refinement of loci associated with lipid levels. Nat Genet (2013) 45(11):1274–83. doi: 10.1038/ng.2797 PMC3838666 24097068

[34] Evangelou E Warren HR Mosen-Ansorena D Mifsud B Pazoki R Gao H. Genetic analysis of over 1 million people identifies 535 new loci associated with blood pressure traits. Nat Genet (2018) 50(10):1412–25. doi: 10.1038/s41588-018-0205-x PMC6284793 30224653

[35] Mahajan A Go MJ Zhang W Below JE Gaulton KJ Ferreira T. Genome-wide trans-ancestry meta-analysis provides insight into the genetic architecture of type 2 diabetes susceptibility. Nat Genet (2014) 46(3):234–44. doi: 10.1038/ng.2897 PMC3969612 24509480

[36] Liu M Jiang Y Wedow R Li Y Brazel DM Chen F. Association studies of up to 1.2 million individuals yield new insights into the genetic etiology of tobacco and alcohol use. Nat Genet (2019) 51(2):237–44. doi: 10.1038/s41588-018-0307-5 PMC6358542 30643251

[37] Li J Tian A Zhu H Chen LL Wen JP Liu WQ. Mendelian randomization analysis reveals no causal relationship between nonalcoholic fatty liver disease and severe COVID-19. Clin Gastroenterol Hepatol (2022) 20(7):1553–60.e78. doi: 10.1016/j.cgh.2022.01.045 35124268 PMC8812093

[38] Jiang L Zheng Z Fang H Yang J. A generalized linear mixed model association tool for biobank-scale data. Nat Genet (2021) 53(11):1616–21. doi: 10.1038/s41588-021-00954-4 34737426

[39] Rizvi S Gores GJ. Pathogenesis, diagnosis, and management of cholangiocarcinoma. Gastroenterology (2013) 145(6):1215–29. doi: 10.1053/j.gastro.2013.10.013 PMC3862291 24140396

[40] Yang B Petrick JL Kelly SP Graubard BI Freedman ND McGlynn KA. Adiposity across the adult life course and incidence of primary liver cancer: the NIH-AARP cohort. Int J Cancer (2017) 141(2):271–8. doi: 10.1002/ijc.30737 PMC5491533 28411388

[41] Chen L Fan Z Sun X Qiu W Mu WT Chai K. Examination on the risk factors of cholangiocarcinoma: a mendelian randomization study. Front Pharmacol (2022) 13:900424. doi: 10.3389/fphar.2022.900424 36091764 PMC9462706

[42] Pearson-Stuttard J Papadimitriou N Markozannes G Cividini S Kakourou A Gill D. Type 2 diabetes and cancer: an umbrella review of observational and mendelian randomization studies. Cancer Epidemiol Biomarkers Prev (2021) 30(6):1218–28. doi: 10.1158/1055-9965.Epi-20-1245 PMC9398112 33737302

[43] Chen HF Chen P Li CY. Risk of malignant neoplasms of liver and biliary tract in diabetic patients with different age and sex stratifications. Hepatology (2010) 52(1):155–63. doi: 10.1002/hep.23641 20578004

[44] Zhou Z Nie SD Jiang B Wang J Lv P. Risk factors for extrahepatic cholangiocarcinoma: a case-control study in China. Eur J Cancer Prev (2019) 28(4):254–7. doi: 10.1097/cej.0000000000000468 30299315

[45] De Lorenzo S Tovoli F Mazzotta A Vasuri F Edeline J Malvi D. Non-alcoholic steatohepatitis as a risk factor for intrahepatic cholangiocarcinoma and its prognostic role. Cancers (Basel) (2020) 12(11):3182. doi: 10.3390/cancers12113182 33138044 PMC7692633

